# Psychiatric disorders in chronic hemodialysis patients in a clinic in Bahia: the influence of the COVID-19 pandemic

**DOI:** 10.1590/2175-8239-JBN-2024-0147en

**Published:** 2025-06-13

**Authors:** Daniela de Queiroz Moura Landim, Melina Pinheiro Gomes de Souza, Lianna Gabriella Dantas, Ana Flávia Moura, José A. Moura-Neto, José Andrade Moura, Constança Margarida Sampaio Cruz

**Affiliations:** 1 Escola Bahiana de Medicina e Saúde Pública, Departamento de Clínica Médica, Salvador, BA, Brazil.; 2 Clínica de Nefrologia de Juazeiro (CLINEFRO), Juazeiro, BA, Brazil.; 3 Instituto de Nefrologia e Hipertensão, Vale do Canela, Salvador, BA, Brazil.; 4 Obras Sociais Irmã Dulce, Salvador, BA, Brazil.

**Keywords:** Mental Disorders, Renal Insufficiency, Chronic, Renal Dialysis, COVID-19

## Abstract

**Introduction:**

Chronic Kidney Disease is associated, due to several factors linked to the disease, with a high rate of psychiatric disorders. With the COVID-19 pandemic, evidence suggests an increase in the already high prevalence of these disorders in chronic kidney patients. It is assumed that early diagnosis of psychiatric disorders can contribute to the development of treatment strategies, reducing the morbidity and mortality caused by the disorders.

**Objective:**

To determine the prevalence of psychiatric disorders in hemodialysis patients, evaluating the association of COVID-19 and some variables with the occurrence of these disorders.

**Methodology:**

Cross-sectional study carried out in a nephrology clinic in Bahia, in 2023. The sample consisted of 119 patients chosen by simple randomization. Patients were evaluated by the Mini International Neuropsychiatric Interview, an internationally validated interview.

**Results:**

Sixty-two patients (52.1%) had at least one psychiatric disorder. The most common were Generalized Anxiety Disorder (GAD) (42%) and Major Depressive Episode (MDE) (18.5%). There was no association between COVID-19 diagnosis and any psychiatric disorder. Among the 58 patients who had COVID-19, men had a lower chance of developing some disorder (OR = 0.30; 95% CI [0.10–0.91]), as did patients with >5 years of treatment (OR = 0.17; 95% CI [0.05–0.61]).

**Conclusions:**

The prevalence of psychiatric disorders is high. Among patients who had COVID-19, women had more psychiatric disorders, as well as, specifically, more GAD, and patients who had more than 5 years of treatment had a significantly lower chance of developing some psychiatric disorder.

## Introduction

Chronic Kidney Disease (CKD) is a major public health issue due to its high prevalence and elevated morbidity and mortality rates. In recent decades, the natural history of this disease has changed with the advent of Renal Replacement Therapy (RRT) techniques^1^, which initially aimed only to increase patient survival. From the early 1960s onwards, these techniques underwent enormous advancements in terms of service quality and accessibility. Currently, in addition to increasing survival rates, efforts are being made to improve the quality of this survival, which is already severely compromised^2^.

Within the current emphasis on quality of life, greater attention has been given to the numerous psychological pressures to which dialysis patients are subjected: a) dependency imposed by treatment; b) fear of death; c) restrictions on fluid intake and diet; d) losses imposed by the disease (unemployment, decline in social status); and e) physical complications of the disease, loss of urinary function, changes in body image, and impaired sexual performance^2^.

Supporting these various factors, the global literature shows that these patients have a high prevalence of psychiatric disorders, mainly depression and anxiety, which are independent predictors of mortality^4^.

In 2019, the world witnessed the emergence of the SARS-CoV-2 coronavirus, the causative agent of COVID-19. Classified as a pandemic in March 2020, the disease led the world to face an unprecedented reality in the modern era: social isolation as a protective measure for life^5^. The consequences have clearly been devastating, both in terms of physical and mental health^5^.

Since then, evidence has shown that patients with comorbidities such as CKD — especially those undergoing hemodialysis — were at higher risk of infection and had worse clinical outcomes^5^, in addition to facing the challenge of adhering to social isolation^5^.

Through the combination of these factors, it is suggested that chronic kidney disease patients may have experienced negative effects on their mental health, with an increase in the already high prevalence of depression and anxiety^5^.

Among the various questionnaires used in prevalence studies, the Mini-International Neuropsychiatric Interview (MINI), validated in Brazil, stands out^6^. Brief and simple to administer, it is useful in identifying these disorders in this specific group of patients^6,7^.

It is believed that a more accurate and early diagnosis of psychiatric disorders may enable significant improvements in the development of prevention and control programs for these disorders, aiming to reduce morbidity and increase patient survival^4^.

For these reasons, the main objective of this study was to determine the prevalence of psychiatric disorders in CKD patients on hemodialysis (HD) during the COVID-19 pandemic, at a clinic in a mid-sized city located inland in the state of Bahia. As secondary objectives, the study aimed to assess the possible association between the diagnosis of COVID-19 and some clinical and sociodemographic variables with the occurrence of psychiatric disorders.

## Method

### Study Design

Cross-sectional cohort study.

### Location and Period

The study was conducted at a satellite nephrology clinic located in Juazeiro, Bahia, between March 2023 and July 2023.

### Sample

**Figure 1:**
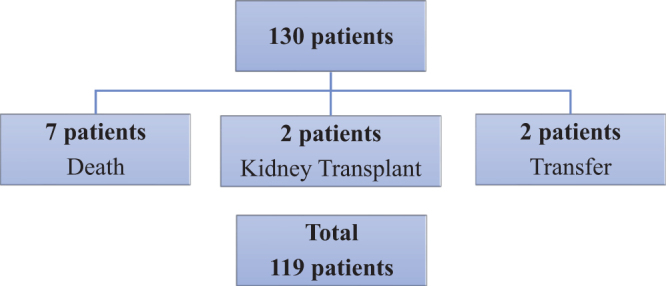
Study inclusion flowchart.

The sample consisted of 119 patients undergoing regular hemodialysis through the Brazilian Unified Health System (SUS) who agreed to participate in the study and signed the Free and Informed Consent Form (FICF). Selection was performed using simple randomization. To determine the sample size, the formula **N = (o** ^2^ **p.q)/e** ^2^, was used, considering **o** ^2^ as the significance level of 5%, **p** as the percentage at which the phenomenon occurs (0.51, assuming that 50% of patients would present some psychiatric disorder)^3,8,9^, **q** as the complementary percentage (49%), and **e** ^2^ as the maximum allowable error of 10%. The study inclusion flowchart is shown in Figure 1.

### Inclusion Criteria

a) Patients ≥ 18 years old;
b) Having advanced-stage CKD and being enrolled in a hemodialysis program at the study site for more than 30 days;
c) Being in stable clinical condition, with no hospitalizations in the last 15 days.

### Exclusion Criteria

a) Patients who did not agree to participate in the study;
b) Patients with cognitive impairments or neurological disorders that prevented the interview;
c) Incomplete questionnaires.

### Ethical Requirements

The study protocol was submitted to the Research Ethics Committee of *Hospital Santo Antônio/Obras Sociais Irmã Dulce*, based on the legal and ethical principles established by Resolution No. 466/2012, of the National Health Council, Brazilian Ministry of Health, and was approved in September 2021 (opinion number: 4.948.336; CAAE: 47873521.6.1001.0047). The researchers ensured patient privacy and anonymity.

### Data Collection and Organization

The interviews were conducted in individual offices by a psychologist trained in the use of MINI, after the patients’ hemodialysis session, with an average application time of 13.2 ± 1.3 minutes.

All questionnaire data were processed electronically using the SPSS Statistics program, version 26.0. Data correction was performed by consistency analysis and searching for recording errors, which were rectified when necessary, by retrieving the questionnaires.

### Study Variables

a) Sex: male and female
b) Age: up to 20 years old; 21 to 40 years old; 41 to 60 years old; over 61 years old.
c) Residence: “rural” (dialysis unit > 50 km from residence); “urban” (residence < 50 km from dialysis center).
d) Marital status: married; single; widowed.
e) COVID: having had COVID or not. Diagnosis was based on positive serology associated with the presence of fever, dyspnea, and/or cough.
f) Time under treatment: < 3 years; between 3 and 5 years; > 5 years.
g) Kt/V: ≤0.80; 0.81 to 1.20; ≥1.21. Calculations were considered to be those made in the month in which the interview was conducted.
h) Hemoglobin: ≤9g/dL; 9.1g/dL to 13g/dL; ≥13.1g/dL (test performed in the month of the interview).

### Mini International Neuropsychiatric Interview (MINI)

This questionnaire was developed in the 1990s for the diagnosis of psychiatric disorders and considers the DSM-III-R/IV (Diagnostic and Statistical Manual of Mental Disorders – 3rd and 4th editions) and the ICD-10 (International Classification of Diseases). It has been validated internationally, as well as for the Portuguese language in Brazil, in 2000^7^. It features a short application time and can be used by individuals after brief training (1 to 3 hours).

It should be noted that a new version of M.I.N.I. is available (7.0.2, based on the updated DSM-V criteria). However, the previous version of the instrument was chosen, as the latest version has not yet been adapted and validated for use in Brazil and for the Portuguese language.

### Statistical Analysis

In the present study, all variables were categorized. In the descriptive analysis, point measures such as frequency and percentages of variables were calculated. The Chi-square and Fisher’s exact tests were used to identify associations between two variables. A significance level of 5% (p < 0.05) was adopted for the statistical tests.

## Results

Of the 550 patients at the studied clinic, 130 were invited to participate in the study, and all agreed and signed the FICF.

After signing the FICF and prior to the interview, 11 patients were unable to continue with the study, as shown in Figure 1. Thus, 119 patients were evaluated, corresponding to 91.5% of the initial sample.

### Sociodemographic and Clinical Variables

Most patients were male (57.1%), aged between 41 and 60 years old (49.6%), lived in urban areas (83.2%), and were married (59.7%).

The proportion of patients who had COVID-19 (48.7%) and those who did not (51.3%) was similar in the sample. The majority had Kt/V > 1.20 (65.5%) and hemoglobin levels between 9.1 and 13.0 g/dL (76.5%). Table 1 illustrates these figures.

**Table 1:** Sociodemographic and clinical variables of patients

Sociodemographic	n	%
Sex		
Female	51	42.9
Male	68	57.1
Age		
≤ 20 years old	0	0
21 to 40 years old	28	23.5
41 to 60 years old	59	49.6
≥ 61 years old	32	26.9
Residence		
Urban	99	83.2
Rural	20	16.8
Marital Status		
Married	71	59.7
Single	38	31.9
Widowed	10	8.4
Clinics	n	%
COVID-19		
Yes	58	48.7
No	61	51.3
Treatment vintage		
≤ 3 years	48	40.3
3 to 5 years	27	227
≥ 5 years	44	37.0
Kt/V		
≤ 0.80	16	13.4
Between 0.81 and 1.20	25	21.1
≥ 1.21	78	65.5
Hemoglobin		
≤ 9 g/dL	10	8.4
Between 9.1 and 13 g/dL	91	76.5
≥ 13.1 g/dL	18	15.1

### Psychiatric Disorders

Of the 119 participants, 18.5% had Major Depressive Episode (MDE) and 42% had Generalized Anxiety Disorder (GAD). These were the main diagnoses identified, and 96.8% of individuals with a diagnosed disorder had either MDE or GAD. Twelve patients (10.1%) presented both associated disorders. Table 2 shows the prevalence rates found in the sample.

**Table 2:** Prevalence of psychiatric disorders in the 119 evaluated subjects

Psychiatric disorders	n (%)
Major Depressive Episode	22 (18.5)
Generalized Anxiety Disorder	50 (42.0)
High risk of suicide	3 (2.5)
Post-Traumatic Stress Disorder	1 (0.8)
Alcohol dependence or abuse	1 (0.8)
Substance dependence or abuse (non-alcohol)	1 (0.8)

The prevalence of patients with only one diagnosis was 39.5%. Fourteen patients (11.8%) had two associated diagnoses, and one (0.8%) had three diagnoses. When considered together, 62 patients (52.1%) were found to have at least one psychiatric disorder.

The following were not diagnosed: dysthymic disorder; hypomanic episode; panic disorder; agoraphobia; social phobia; obsessive-compulsive disorder; psychotic syndrome; anorexia nervosa; bulimia nervosa, or antisocial personality disorder.

### Variables Associated with Psychiatric Disorders

#### Diagnosis of COVID-19

The mean time between COVID-19 diagnosis and the interview was 132 days, with a maximum time of approximately 300 days.

The subgroup of patients who had COVID-19 and the subgroup of those who did not were similar, and the diagnosis of COVID-19 had no impact on the likelihood of an individual presenting with a psychiatric disorder, as shown in Table 3.

**Table 3:** Association between COVID-19 diagnosis and psychiatric disorders

Any psychiatric disorder	No n (%)	Yes n (%)	p-value
No COVID-19	30 (52.6)	31 (50.0)	0.774
COVID-19	27 (47.4)	31 (50.0)	
Major Depressive Episode	No n (%)	Yes n (%)	p-value
No COVID-19	49 (50.5)	12 (54.5)	0.733
COVID-19	48 (49.5)	10 (45.5)	
Generalized Anxiety Disorder	No n (%)	Yes n (%)	p-value
No COVID-19	37 (53.6)	24 (48.0)	0.545
COVID-19	32 (46.4)	26 (52.0)	

When evaluating the subgroup of 58 patients who had COVID-19, it was observed that, within this group, female sex predisposed to the diagnosis of some psychiatric disorder (p = 0.03). In the bivariate analysis, considering women as reference, men were less than half as likely to develop a psychiatric disorder compared to women (OR = 0.30; 95% CI [0.10–0.91]). Similarly, patients with < 3 years on dialysis treatment had more diagnosed psychiatric disorders (58.1%; p = 0.017). Bivariate analysis showed that patients with > 5 years of treatment had a significantly lower chance of presenting any psychiatric disorder (OR = 0.17; 95% CI [0.05–0.61]). These data are shown in Table 4.

**Table 4:** Association between clinical and sociodemographic data and psychiatric disorders in the 58 participants who had COVID-19

Variables	No psychiatric disorder n (%)	With psychiatric disorder n (%)	p-value*	OR [95% CI]
Sex			0.030	
Female	8 (29.6)	18 (58.1)		–
Male	19 (70.4)	13 (41.9)		0.30 (0.10–0.91)
Age			0.585	
21 to 40 years old	8 (29.6)	7 (22.6)		–
41 to 60 years old	12 (44.4)	18 (58.1)		1.71 (0.49–5.98)
≥ 61 years old	7 (25.9)	6 (19.4)		0.98 (0.22–4.34)
Residence			0.435**	
Urban	24 (88.9)	26 (83.9)		–
Rural	3 (11.1)	5 (16.1)		1.54 (0.33–7.14)
Marital Status			0.320	
Married	14 (51.9)	21 (67.7)		–
Single	10 (37.0)	6 (19.4)		0.40 (0.12–1.35)
Widowed	3 (11.1)	4 (12.9)		0.89 (0.17–4.59)
Treatment vintage			0.017	
≤ 3 years	6 (22.2)	18 (58.1)		–
Between 3 and 5 years	7 (25.9)	6 (19.4)		0.29 (0.07–1.19)
≥ 5 years	14 (51.9)	7 (22.6)		0.17 (0.05–0.61)
KT/V			0.555	
≤ 0.80	5 (18.5)	3 (9.7)		–
Between 0.81 and 1.20	6 (22.2)	6 (19.4)		1.67 (0.27–10.33)
≥ 1.21	16 (59.3)	22 (71.0)		2.29 (0.48–11.01)
Hemoglobin			0.786	
≤ 9 g/dL	3 (11.1)	2 (6.5)		–
Between 9.1 and 13 g/dL	20 (74.1)	25 (80.6)		3.50 (0.36–33.82)
≥ 13.1 g/dL	4 (14.8)	4 (12.9)		4.00 (0.30–53.47)

*Chi-square test;

**Fisher&apos;s exact test.

When specific disorders were evaluated, the analysis showed that women (p = 0.021) and patients with up to 3 years of treatment (p = 0.002) in this subgroup were more likely to develop GAD. Thus, men had a 0.28 times lower chance of developing GAD (OR = 0.28; 95% CI [0.09–0.84]), and patients with more than 5 years of treatment had a 0.10 times lower chance of developing GAD (OR = 0.10; 95% CI [0.02–0.39]).

#### Sociodemographic and Clinical Variables

When correlating the other sociodemographic and clinical variables with the prevalence of Major Depressive Episode, it was observed that no association presented statistical power.

However, when specifically evaluating GAD, it was found that 54% of patients with this disorder had up to 3 years of treatment, compared to 30.4% of patients with no GAD (p = 0.029). In the bivariate analysis, patients with > 5 years of treatment had a lower risk of developing any psychiatric disorder (OR = 0.33; 95% CI [0.14–0.77]). These data can be seen in Table 5.

**Table 5:** Association between clinical and sociodemographic data and Generalized Anxiety Disorder in 119 evaluated subjects

Variables	No GAD n (%)	GAD n (%)	p-value*	OR [95% CI]
Sex			0.004	
Female	22 (31.9)	29 (58.0)		–
Male	47 (68.1)	21 (42.0)		0.34 (0.16–0.72)
Age			0.472	
21 to 40 years old	19 (27.5)	9 (18.0)		–
41 to 60 years old	32 (46.4)	27 (54.0)		1.78 (0.69–4.58)
≥ 61 years old	18 (26.1)	14 (28.0)		1.64 (0.57–4.72)
Residence			0.233	
Urban	55 (79.7)	44 (88.0)		–
Rural	14 (20.3)	6 (12.0)		0.54 (0.19–1.51)
Marital Status			0.461	
Married	38 (55.1)	33 (66.0)		–
Single	25 (36.2)	13 (26.0)		0.60 (0.26–1.35)
Widowed	6 (8.7)	4 (8.0)		0.77 (0.20–2.96)
Treatment vintage			0.029	
≤ 3 years	21 (30.4)	27 (54.0)		–
Between 3 and 5 years	17 (24.6)	10 (20.0)		0.46 (0.17–1.20)
≥ 5 years	31 (44.9)	13 (26.0)		0.33 (0.14–0.77)
KT/V			0.332	
≤ 0.80	12 (17.4)	4 (8.0)		–
Between 0.81 and 1.20	14 (20.3)	11 (22.0)		2.36 (0.59–9.37)
≥ 1.21	43 (62.3)	35 (70.0)		2.44 (0.72–8.24)
Hemoglobin			0.300	
≤ 9 g/dL	8 (11.6)	2 (4.0)		–
Between 9.1 and 13 g/dL	50 (72.5)	41 (82.0)		3.28 (0.66–16.30)
≥ 13.1 g/dL	11 (15.9)	7 (14.0)		2.54 (0.41–15.65)

Note – *Chi-square test.

## Discussion

The psychosocial difficulties presented by CKD patients, particularly those undergoing chronic dialysis, have been the subject of growing interest in recent decades. Several studies have demonstrated that the high prevalence of psychiatric disorders in this population^3,10^ is directly related to the patient’s reduced ability to maintain dialysis treatment and to a worse prognosis, with longer hospitalizations and reduced survival in this population^3,4,11–13^.

In addition, there is increasing emphasis on the quality of life of these individuals, with psychiatric disorders being one of the main factors contributing to poor quality of life in these patients^11–13^. The analysis and study of these disorders in this population enable the development of specific treatment strategies aimed at increasing survival and improving quality of life^13^.

Unfortunately, it is difficult to determine the actual prevalence of psychiatric disorders in this group of patients. The potential confusion between symptoms characteristic of psychiatric disorders and symptoms of uremia contributes to this fact. Irritability, insomnia, loss of appetite, fatigue, loss of libido, and difficulty concentrating are some of the symptoms that may be attributed to anxiety and depression, and which may also be present in chronic kidney disease patients due to disease-specific factors^14^.

Moreover, uremic toxins directly contribute to brain damage and consequent cognitive decline and psychiatric disorders, as well as hemodynamic changes, anemia, hyperparathyroidism, polypharmacy, and sleep disorders^3^.

The use of questionnaires in studies for the diagnosis of disorders is another factor that may alter the actual prevalence of the diseases, with a tendency to overestimate their value. This fact was observed in a 1985 study, in which depression was identified in 47% of HD patients following the application of the Beck Depression Inventory (BDI). However, after evaluation using more rigorous methods, it was shown that only 5% of these patients actually met the DSM-III criteria for depression^11^.

Even considering the possibility of false-positive diagnoses, the detection of potential disorders will enable greater attention and commitment from the healthcare team, remedying the underestimation of these disorders in chronic dialysis services.

The MINI 5.0.0. is a questionnaire that offers several advantages for its use. One of the factors that supported the decision to use it was the existence of a previous study, published in 2006, evaluating psychiatric disorders in CKD patients in Bahia^15^, which facilitates the comparison of the prevalence of these disorders over the years and after the COVID-19 pandemic.

In the present study, a significant percentage of the sample exhibited psychiatric disorders. This confirms that high rates of such disorders occur in hemodialysis units, more than double the rate reported by the Ministry of Health for the general population in Brazil, which indicates a prevalence of mental disorders of around 20% in the adult population^16^. Internationally, a study published in 2023, which aggregated data from 29 countries and included 156,331 respondents, demonstrated that the lifetime prevalence of mental disorders was approximately 29%, with the main disorders being alcohol abuse, major depressive disorder, and specific phobia^17^.

In the current study, the most frequent disorders identified were Major Depressive Episode, either current or recurrent, in 18.5% of patients, and Generalized Anxiety Disorder, in 42%. These data far exceed the rates found in the general Latin American population, estimated at around 8.7% for MDE and 5.5% for GAD^18^. Among adults from São Paulo, studies indicate a prevalence of 11% for mood disorders and 19.9% for anxiety disorders^19^.

In Brazil, two previous studies used the MINI in chronic kidney disease patients. The first, conducted 20 years ago, evaluated 244 dialysis patients in Bahia^15^. This study revealed a prevalence of 37.3% for psychiatric disorders, with 8.6% for MDE and 2% for GAD^15^. The second study, conducted in 2009, assessed only mood and psychotic disorders using the MINI. Seventy HD patients in Rio Grande do Sul were interviewed, and the prevalence of major depression found was 9.9%^20^.

Several factors may explain the increase in the prevalence of these disorders over the last few decades, such as: an aging population and an increase in chronic diseases; high external and self-imposed demands; difficulty in coping with challenging situations; growing exposure to violence and crime; and the advent of the COVID-19 pandemic and its consequences^21^.

Even though the use of a different diagnostic method hinders comparison with the current study, it is worth mentioning a study conducted in the United States in 1993, which evaluated 93% of all dialysis patients in the country and reported that approximately 9% of these patients had at least one hospitalization for a primary or secondary diagnosis of a mental disorder during the study year^22^. In addition, a meta-analysis evaluated 55,982 people with CKD worldwide, showing a prevalence ranging from 22.8% to 39.3%, depending on the method used^10^. It is worth noting that this study assessed depressive symptoms in general, and not Major Depressive Episode alone.

As previously mentioned, the advent of the COVID-19 pandemic has further increased the high prevalence of psychiatric disorders, with rates increasing to around 27.6% for MDE and 25.6% for GAD^9,21^. During active infection, the prevalence is even higher, with 45% of patients presenting with depression and 47% with anxiety^9^. It is believed that, in addition to the factors inherent to the infection and social isolation, concern for family and friends, uncertainty about the future, inaccurate information disseminated on social media^23^, and shortage of medical supplies contributed to this increase.

Due to the suspicion of the existence of these different contributors and in an attempt to assess the isolated contribution of infection-related factors, the present study divided the interviewees into two sociodemographically similar subgroups: those who did not have COVID-19 and those who had the infection. Contrary to expectations, no statistically significant association was found between a diagnosis of COVID-19 and an increased prevalence of disorders. One factor that may partly explain this finding is that the study patients were those presenting with mild infection. Many dialysis patients who developed severe COVID-19 progressed to death and therefore could not be included in this study.

Among the sociodemographic variables analyzed, female sex confirmed data already described in the literature^24–26^, showing a positive association with the occurrence of mental disorders.

The association was more evident and statistically significant when directed toward the diagnosis of GAD or when the analysis was performed specifically in the subgroup of 58 patients who had COVID-19. This is likely due to the higher number of patients diagnosed with GAD (50 patients) compared to MDE (22 patients) and, perhaps, to the fact that, although there was no significant association between COVID-19 diagnosis and psychiatric disorders, the prevalence of women with psychiatric disorders in this group was higher (58.1% versus 45.2%).

Thus, women were twice as likely as men to develop a psychiatric disorder, more specifically GAD. These data reinforce the assumption that women have greater difficulty coping with adverse situations, in addition to having intrinsic personality traits that are more susceptible to changes in mental health when confronted with chronic health problems^26^.

Contrary to what some studies in the literature suggest, no association was found between place of residence, marital status, and its consequent family support^27^. This may have occurred because this study only assessed the patient’s place of residence and marital status, without questioning whether they had any type of family support beyond their spouse or even any social support outside this context.

With regard to age in particular, it is noteworthy that there were no patients under the age of 20 in the sample, and that the majority of patients (73.1%) were between 20 and 60 years old. This epidemiological profile is similar to that observed in units in other countries, where the highest prevalence of dialysis is in patients between 20 and 59 years old^28^. However, this characteristic may have contributed to the lack of association, since older adults have comorbidities that reflect poorer quality of life and greater expression of psychiatric disorders^29,30^. In addition, some studies show that younger individuals have greater difficulty coping with chronic diseases, presenting higher rates of psychiatric disorders when these diseases, such as CKD, are diagnosed^24^.

Another variable that showed an association with psychiatric disorders was the length of treatment for the disease. Patients who had been on hemodialysis for less than 3 years exhibited more disorders, suggesting that a longer time on a dialysis program was predictive of better adaptation to treatment, a finding consistent with most data in the literature^31^. A 1987 study demonstrated a higher prevalence of depression within the first 2 years of treatment^32^. CKD patients often experience improvements in their medical conditions after initiating dialysis, but they require some time to psychologically adapt to their new situation^31^. However, there is no consensus on this issue. Kimmel et al., in 1998, evaluated hospitalizations for mental illness in 176,368 dialysis patients. In that study, time on dialysis was not associated with an increased risk of hospitalization^22^.

The presence of anemia did not increase the risk of patients expressing psychiatric disorders, although higher hemoglobin levels were positively correlated with better quality of life and improved symptoms^33^. Our results may be due to the small number of patients with hemoglobin levels below 9 g/dL.

Similarly, no association was found between Kt/V and psychiatric disorders. This is again believed to be due to the fact that most of the patients studied had a Kt/V greater than 1.21 (target value)^34^.

### Limitations

Some limitations were related to the impracticality of assessing all variables of importance, which would undoubtedly have added greater validity to the study. Among these, patients’ personality traits and their perception of the disease are reported as important predictors of mental health^35^, as are the etiology of CKD, associated comorbidities, race, occupation, family income, educational level, family support, nutritional status, and hormonal changes^24,25,31,35^.

Additionally, it is believed that the sample size was insufficient to give significance to some of the differences observed. A prevalence of 50% of psychiatric disorders was used to calculate the sample size, which was similar to the rate found in the present study. However, if a lower prevalence had been used for the calculation, the sample size would have been larger, which would have increased the significance of the findings.

The difficulty in comparing the present study with other population-based studies may also be considered a limitation, as this is a single-center study and the MINI questionnaire used was an older version, still based on the DSM-III-R/IV criteria, given that it was the only version validated for the Portuguese language^7^.

It should be remembered that there is a risk of confusion between symptoms characteristic of these disorders and organic factors inherent to CKD^4,14^. In the present study, dialysis effectiveness was assessed using Kt/V and interviews were conducted after hemodialysis sessions, in an attempt to minimize this potential confusion. However, it is known that there are other clinical and laboratory variables that could contribute to the assessment of dialysis adequacy that were not used in this study.

Finally, even though the sample in this study is representative of HD patients in Bahia, comparisons with other units in Brazil and around the world are more problematic, as they present a distinct epidemiological profile^36^.

### Clinical Implications

Psychiatric disorders are important and independent predictors of morbidity and mortality in dialysis, mainly because they are associated with non-adherence to treatment^3,4,11,12^. In a 2000 meta-analysis involving more than 500 patients, the authors concluded that the risk of non-adherence to treatment in depressed patients was three times higher than in non-depressed patients^37^.

We believe that research on psychosomatic, psychiatric, and quality-of-life aspects could make an important contribution to clinical decision-making and patient care in CKD. Studies have confirmed the role of these psychosomatic aspects in influencing morbidity and mortality in these patients^13,37^.

Despite their high prevalence and significant clinical and socioeconomic burden, psychiatric disorders appear to be undertreated in CKD patients. A large study involving 1,099 adults with CKD who exhibited depressive symptoms revealed that only 31% of patients reported having been prescribed antidepressants^37^.

As in the general population, treatment of these disorders should include nonpharmacological strategies such as psychotherapy, physical training programs, and social support^38–40^. Studies have demonstrated that physical exercise programs may reduce depressive symptoms in dialysis patients^38,39^. A randomized trial involving 85 hemodialysis patients with depressive symptoms showed significant improvement after 12 weeks of cognitive-behavioral therapy^40^.

It is anticipated that the data and results described herein will stimulate further research in this area, raise awareness of the extremely high prevalence of these disorders within dialysis units, and ultimately contribute in some way to improving the quality of life and survival of CKD patients undergoing hemodialysis.

## Conclusions

The prevalence of psychiatric disorders among CKD patients undergoing chronic hemodialysis at an RRT clinic in an inland area of Bahia was high, reaching 52.1%. The most common disorders were Generalized Anxiety Disorder and Major Depressive Episode, with almost all patients (96.8%) presenting at least one of these two diagnoses.

The diagnosis of COVID-19 showed no positive association with the occurrence of any psychiatric disorder. However, among the subgroup of patients who had COVID-19, women presented significantly higher rates of psychiatric disorders, while patients with more than 5 years of treatment had a significantly lower chance of developing any disorder, compared to the others.

## Data Availability

The datasets generated and analyzed during the current study are not publicly available due to ethical and privacy restrictions but are available from the corresponding author upon reasonable request.
