# Supplementary material for: Psychiatric disorders in chronic hemodialysis patients in a clinic in Bahia: the influence of the COVID-19 pandemic
Source: J Bras Nefrol. 2025 Jun 13;47(3):e20240147. doi: 10.1590/2175-8239-JBN-2024-0147en (PMC12221274; doi:10.1590/2175-8239-JBN-2024-0147en)
Supplement: Translation

# Translation: Transtornos psiquiátricos em pacientes em hemodiálise crônica em uma clínica na Bahia: a influência da pandemia de COVID-19

**DOI:** 10.1590/2175-8239-JBN-2024-0147pt

**Authors:** Daniela de Queiroz Moura Landim, Melina Pinheiro Gomes de Souza, Lianna Gabriella Dantas, Ana Flávia Moura, José A. Moura-Neto, José Andrade Moura, Constança Margarida Sampaio Cruz

**Affiliations:** 1 Escola Bahiana de Medicina e Saúde Pública, Departamento de Clínica Médica, Salvador, BA, Brasil.; 2 Clínica de Nefrologia de Juazeiro (CLINEFRO), Juazeiro, BA, Brasil.; 3 Instituto de Nefrologia e Hipertensão, Vale do Canela, Salvador, BA, Brasil.; 4 Obras Sociais Irmã Dulce, Salvador, BA, Brasil.

**Keywords:** Transtornos Mentais, Insuficiência Renal Crônica, Diálise Renal, COVID-19

## Abstract

**Introdução:**

A Doença Renal Crônica está associada, por diversos fatores ligados à doença, a uma alta taxa de distúrbios psiquiátricos. Com a pandemia da COVID-19, evidências sugerem um aumento da já elevada prevalência desses distúrbios em pacientes renais crônicos. Admite-se que o diagnóstico precoce de transtornos psiquiátricos possa contribuir para a elaboração de estratégias de tratamento, diminuindo a morbimortalidade causada pelos transtornos.

**Objetivo:**

Determinar a prevalência de distúrbios psiquiátricos em pacientes em hemodiálise, avaliando a associação da COVID-19 e de algumas variáveis com a ocorrência desses transtornos.

**Metodologia:**

Estudo de corte transversal realizado em uma clínica de nefrologia na Bahia, em 2023. A amostra foi composta por 119 pacientes selecionados por randomização simples. Os pacientes foram avaliados pelo *Mini International Neuropsychiatric Interview*, entrevista validada internacionalmente.

**Resultados:**

Sessenta e dois pacientes (52,1%) apresentaram ao menos um transtorno psiquiátrico. Os mais comuns foram Transtorno de Ansiedade Generalizada (TAG) (42%) e Episódio Depressivo Maior (EDM) (18,5%). Não houve associação do diagnóstico de COVID-19 com qualquer distúrbio psiquiátrico. Entre os 58 pacientes que tiveram COVID-19, os homens apresentaram menor chance de desenvolver algum transtorno (OR = 0,30; IC 95% [0,10–0,91]), assim como os pacientes com >5 anos de tratamento (OR = 0,17; IC 95% [0,05–0,61]).

**Conclusões:**

A prevalência de transtornos psiquiátricos é elevada. Entre os pacientes que tiveram COVID-19, as mulheres apresentaram mais distúrbios psiquiátricos, bem como mais TAG especificamente. Além disso, os pacientes com mais de 5 anos de tratamento apresentaram uma chance significantemente menor de desenvolver algum transtorno psiquiátrico.

## Introdução

A Doença Renal Crônica (DRC) é um relevante problema na saúde pública por sua elevada prevalência e alta morbimortalidade. Há poucas décadas, a história natural dessa doença modificou-se com o surgimento das técnicas de Terapia Renal Substitutiva (TRS)^1^, que, em seu início, objetivavam apenas aumentar a sobrevida dos pacientes. A partir do início dos anos sessenta, essas técnicas experimentaram enorme avanço na qualidade e democratização dos serviços. Atualmente, busca-se, além do aumento da sobrevida, a melhora na qualidade dessa sobrevida, já bastante comprometida^2^.

Dentro da atual ênfase nessa qualidade de vida, dá-se uma maior atenção às inúmeras pressões psicológicas a que os pacientes em diálise são submetidos: a) dependência imposta pelo tratamento; b) medo da morte; c) restrições à ingesta hídrica e à dieta; d) perdas impostas pela patologia (desemprego, queda de status social); e) complicações físicas da doença, perda da capacidade de urinar, mudanças na imagem corporal e na performance sexual^2^.

Corroborando com esses diversos fatores, a literatura mundial demonstra que esses pacientes apresentam uma alta prevalência de distúrbios psiquiátricos, principalmente depressão e ansiedade^3^, sendo estes preditores independentes de mortalidade^4^.

Em 2019 o mundo presenciou o surgimento do coronavírus SARS-CoV-2, causador da COVID-19. Classificada como pandemia em março de 2020, a doença levou o mundo a enfrentar uma realidade sem precedentes na era moderna: o isolamento social como medida protetiva de vida^5^. Claramente, as consequências foram devastadoras na esfera da saúde física e mental^5^.

Desde então, evidências mostraram que pacientes com comorbidades, como a DRC - especialmente aqueles em programa de hemodiálise - apresentavam maior risco de infecção e pior desfecho clínico da doença^5^, além de se depararem com o desafio da aderência ao isolamento^5^.

Através da junção de tais fatores, sugere-se que pacientes renais crônicos possam ter evoluído com efeitos negativos em sua saúde mental, com aumento da prevalência, já antes elevada, de depressão e ansiedade^5^.

Entre os diversos questionários utilizados em estudos de prevalência, destaca-se o Mini-International Neuropsychiatric Interview (MINI), validado no Brasil^6^. Breve e simples em sua aplicação, é útil na identificação desses transtornos nesse grupo específico de pacientes^6,7^.

Acredita-se que um diagnóstico mais preciso e precoce dos transtornos psiquiátricos possa possibilitar melhorias significativas na elaboração de programas de prevenção e controle desses transtornos, visando à diminuição da morbidade e ao aumento da sobrevida dos pacientes^4^.

Por essas razões, o objetivo principal deste trabalho foi determinar a prevalência de distúrbios psiquiátricos em pacientes com DRC em hemodiálise (HD) durante a pandemia da COVID-19, em uma clínica no interior da Bahia. Como objetivos secundários, buscou-se avaliar a possível associação do diagnóstico de COVID-19 e de algumas variáveis clínicas e sociodemográficas com a ocorrência dos transtornos psiquiátricos.

## Método

### Desenho do Estudo

Estudo de coorte transversal.

### Localização e Período

A pesquisa foi realizada em uma clínica satélite de nefrologia, localizada em Juazeiro, Bahia, entre março de 2023 e julho de 2023.

### Amostra

A amostra foi constituída por 119 pacientes em hemodiálise regular pelo SUS, que concordaram em participar do estudo e assinaram o Termo de Consentimento Livre e Esclarecido (TCLE). A seleção foi realizada por randomização simples. Para definir o tamanho da amostra, utilizou-se a fórmula **N = (o** ^2^ **p.q)/e** ^2^, considerando-se **o** ^2^ como o nível de significância de 5%, **p** como a porcentagem com a qual o fenômeno se verifica (0,51, assumindo-se que 50% dos pacientes apresentariam algum distúrbio psiquiátrico)^3,8,9^, **q** como a porcentagem complementar (49%) e **e** ^2^ como o erro máximo permitido, de 10%. O fluxograma de inclusão no estudo é apresentado na Figura 1.

**Figura 1:** Fluxograma de inclusão no estudo.

### Critérios de Inclusão

a) Pacientes ≥ 18 anos;
b) Ser portador de DRC em estágio avançado e estar em programa de hemodiálise na unidade do estudo há mais de 30 dias;
c) Encontrar-se em condições clínicas estáveis, sem internações nos últimos 15 dias.

### Critérios de Exclusão

a) Pacientes que não aceitaram participar do estudo;
b) Pacientes com limitações cognitivas ou desordens neurológicas que impossibilitassem a entrevista;
c) Questionários incompletos.

### Requisitos éticos

O protocolo do estudo foi submetido ao Comitê de Ética em Pesquisa do Hospital Santo Antônio/Obras Sociais Irmã Dulce, com base nos princípios legais e éticos estabelecidos na Resolução nº 466/2012, do Conselho Nacional de Saúde, do Ministério da Saúde do Brasil, tendo sido aprovado em setembro de 2021 (número do parecer: 4.948.336; CAAE: 47873521.6.1001.0047). Os pesquisadores preservaram a privacidade dos pacientes, assegurando seu anonimato.

### Coleta e Organização dos Dados

As entrevistas foram realizadas em consultórios individuais por uma psicóloga treinada na utilização do MINI, após a sessão de hemodiálise dos pacientes, com tempo médio de aplicação de 13,2 ± 1,3 minutos.

Todos os dados do questionário foram processados em base eletrônica por meio do programa SPSS Statistics, versão 26.0. A correção dos dados foi realizada por análise de consistência e busca de erros de registro, os quais foram retificados, quando encontrados, por meio da recuperação dos questionários.

### Variáveis do Estudo

a) Sexo: masculino e feminino
b) Idade: até 20 anos; de 21 a 40 anos; de 41 a 60 anos; superior a 61 anos.
c) Moradia: “rural” (unidade de diálise > 50 km da residência); “urbana” (domicílio < 50 km do centro de diálise).
d) Estado civil: casado; solteiro; viúvo.
e) COVID: ter tido COVID; não ter tido. O diagnóstico foi baseado em sorologia positiva associada à febre, dispneia e/ou tosse.
f) Tempo em tratamento: < 3 anos; entre 3 e 5 anos; > 5 anos.
g) Kt/V: ≤0,80; 0,81 a 1,20; ≥1,21. Considerou-se o cálculo realizado no mês em que foi realizada a entrevista.
h) Hemoglobina: ≤9g/dL; 9,1g/dL a 13g/dL; ≥13,1g/dL (exame realizado no mês da entrevista).

### Mini International Neuropsychiatric Interview (MINI)

Esse questionário foi desenvolvido na década de 1990 para o diagnóstico de transtornos psiquiátricos, e considera o DSM-III-R/IV (Manual Diagnóstico e Estatístico de Transtornos Mentais – 3^a^ e 4^a^ edições) e a CID-10 (Classificação Internacional de Doenças), sendo validado internacionalmente e também para a língua portuguesa no Brasil, em 2000^7^. Apresenta curto tempo de aplicação e a possibilidade de ser utilizado por pessoas após treinamento prévio rápido (1 a 3 horas).

Deve-se ressaltar que existe uma nova versão do M.I.N.I. (7.0.2 com base nos critérios atualizados do DSM-V). Optou-se, entretanto, pela escolha da versão anterior do instrumento, uma vez que a mais recente ainda não foi adaptada e validada para uso no Brasil e em língua portuguesa.

### Análise Estatística

No presente estudo, todas as variáveis foram categorizadas. Na análise descritiva, foram calculadas medidas pontuais, como frequência e percentuais das variáveis. Os testes de Qui-Quadrado e Exato de Fisher foram utilizados para identificar associação entre duas variáveis. O nível de significância adotado nos testes estatísticos foi de 5% (p < 0,05).

## Resultados

Do total de 550 pacientes na clínica estudada, 130 foram convidados a participar do estudo, e todos concordaram e assinaram o TCLE.

Após a assinatura do TCLE e antes da entrevista, 11 pacientes ficaram impossibilitados de seguir com o estudo, conforme mostra a Figura 1. Dessa forma, 119 pacientes foram avaliados, correspondendo a 91,5% da amostra inicial.

### Variáveis Sociodemográficas e Clínicas

A maioria dos pacientes era do sexo masculino (57,1%), tinha entre 41 e 60 anos (49,6%), residia em área urbana (83,2%) e era casada (59,7%).

A proporção de pacientes que tiveram COVID-19 (48,7%) e os que não tiveram (51,3%) foi semelhante na amostra. A maioria apresentou Kt/V > 1,20 (65,5%) e hemoglobina entre 9,1 e 13,0 g/dL (76,5%). A Tabela 1 ilustra esses números.

**Tabela 1:** Variáveis sociodemográficas e clínicas dos pacientes

Sociodemográficas	n	%
Sexo		
Feminino	51	42,9
Masculino	68	57,1
Idade		
≤ 20 anos	0	0
De 21 a 40 anos	28	23,5
De 41 a 60 anos	59	49,6
≥ 61 anos	32	26,9
Moradia		
Urbana	99	83,2
Rural	20	16,8
Estado Civil		
Casado(a)	71	59,7
Solteiro(a)	38	31,9
Viúvo(a)	10	8,4
Clínicas	n	%
COVID-19		
Sim	58	48,7
Não	61	51,3
Tempo de tratamento		
≤ 3 anos	48	40,3
Entre 3 e 5 anos	27	22,7
≥ 5 anos	44	37,0
Kt/V		
≤ 0,80	16	13,4
Entre 0,81 e 1,20	25	21,1
≥ 1,21	78	65,5
Hemoglobina		
≤ 9 g/dL	10	8,4
Entre 9,1 e 13 g/dL	91	76,5
≥ 13,1 g/dL	18	15,1

### Distúrbios Psiquiátricos

Dos 119 participantes, 18,5% apresentavam Episódio Depressivo Maior (EDM) e 42% tinham Transtorno de Ansiedade Generalizada (TAG). Esses foram os principais diagnósticos encontrados e 96,8% das pessoas com algum transtorno diagnosticado tinham EDM ou TAG. Doze pacientes (10,1%) apresentaram as duas patologias associadas. A Tabela 2 apresenta as taxas de prevalência encontradas na amostra.

**Tabela 2:** Prevalência de transtornos psiquiátricos nos 119 participantes avaliados

Distúrbios psiquiátricos	n (%)
Episódio Depressivo Maior	22 (18,5)
Transtorno de Ansiedade Generalizada	50 (42,0)
Risco elevado de suicídio	3 (2,5)
Transtorno de Estresse Pós-Traumático	1 (0,8)
Dependência ou abuso de álcool	1 (0,8)
Dependência ou abuso de substância (não álcool)	1 (0,8)

A prevalência de pacientes com apenas um diagnóstico foi de 39,5%. Quatorze pacientes (11,8%) tiveram dois diagnósticos associados e um (0,8%) apresentou três diagnósticos. Quando considerados em conjunto, 62 pacientes (52,1%) apresentaram algum distúrbio psiquiátrico.

Não foram diagnosticados: transtorno distímico; episódio hipomaníaco; transtorno de pânico; agorafobia; fobia social; transtorno obsessivo-compulsivo; síndrome psicótica; anorexia nervosa; bulimia nervosa ou transtorno de personalidade antissocial.

### Variáveis Associadas aos Transtornos Psiquiátricos

#### Diagnóstico de COVID-19

O tempo médio entre o diagnóstico da COVID-19 e a realização da entrevista foi de 132 dias, sendo o tempo máximo de aproximadamente 300 dias.

O subgrupo de pacientes que tiveram COVID-19 e o subgrupo dos que não tiveram foram semelhantes entre si, e o diagnóstico da COVID-19 não representou impacto algum na probabilidade de o indivíduo apresentar ou não algum transtorno psiquiátrico, conforme demonstrado na Tabela 3.

**Tabela 3:** Associação entre o diagnóstico de COVID-19 e distúrbios psiquiátricos

Algum distúrbio psiquiátrico	Não n (%)	Sim n (%)	p-valor
Não teve COVID-19	30 (52,6)	31 (50,0)	0,774
Teve COVID-19	27 (47,4)	31 (50,0)	
Episódio Depressivo Maior	Não n (%)	Sim n (%)	p-valor
Não teve COVID-19	49 (50,5)	12 (54,5)	0,733
Teve COVID-19	48 (49,5)	10 (45,5)	
Transtorno de Ansiedade Generalizada	Não n (%)	Sim n (%)	p-valor
Não teve COVID-19	37 (53,6)	24 (48,0)	0,545
Teve COVID-19	32 (46,4)	26 (52,0)	

Quando realizada a avaliação do subgrupo de 58 pacientes que tiveram COVID-19, observou-se que, nesse grupo, o sexo feminino predispôs ao diagnóstico de algum distúrbio psiquiátrico (p = 0,03). Na análise bivariada, considerando as mulheres como referência, os homens apresentaram menos da metade da probabilidade de desenvolver algum transtorno psiquiátrico em relação às mulheres (OR = 0,30; IC 95% [0,10–0,91]). Da mesma forma, pacientes com < 3 anos em tratamento dialítico tiveram mais distúrbios psiquiátricos diagnosticados (58,1%; p = 0,017). A análise bivariada mostrou que pacientes com > 5 anos de tratamento tiveram uma chance significantemente menor de apresentar algum transtorno psiquiátrico (OR = 0,17; IC 95% [0,05–0,61]). Esses dados estão demonstrados na Tabela 4.

**Tabela 4:** Associação entre dados clínicos e sociodemográficos e distúrbios psiquiátricos nos 58 participantes que tiveram COVID-19

Variáveis	Sem Dist. Psiq. n (%)	Com Dist. Psiq. n (%)	p-valor*	OR [I.C. 95%]
Sexo			0,030	
Feminino	8 (29,6)	18 (58,1)		–
Masculino	19 (70,4)	13 (41,9)		0,30 (0,10–0,91)
Idade			0,585	
De 21 a 40 anos	8 (29,6)	7 (22,6)		–
De 41 a 60 anos	12 (44,4)	18 (58,1)		1,71 (0,49–5,98)
≥ 61 anos	7 (25,9)	6 (19,4)		0,98 (0,22–4,34)
Moradia			0,435**	
Urbana	24 (88,9)	26 (83,9)		–
Rural	3 (11,1)	5 (16,1)		1,54 (0,33–7,14)
Estado Civil			0,320	
Casado(a)	14 (51,9)	21 (67,7)		–
Solteiro(a)	10 (37,0)	6 (19,4)		0,40 (0,12–1,35)
Viúvo(a)	3 (11,1)	4 (12,9)		0,89 (0,17–4,59)
Tempo de tratamento			0,017	
≤ 3 anos	6 (22,2)	18 (58,1)		–
Entre 3 e 5 anos	7 (25,9)	6 (19,4)		0,29 (0,07–1,19)
≥ 5 anos	14 (51,9)	7 (22,6)		0,17 (0,05–0,61)
KT/V			0,555	
≤ 0,80	5 (18,5)	3 (9,7)		–
Entre 0,81 e 1,20	6 (22,2)	6 (19,4)		1,67 (0,27–10,33)
≥ 1,21	16 (59,3)	22 (71,0)		2,29 (0,48–11,01)
Hemoglobina			0,786	
≤ 9 g/dL	3 (11,1)	2 (6,5)		–
Entre 9,1 e 13 g/dL	20 (74,1)	25 (80,6)		3,50 (0,36–33,82)
≥ 13,1 g/dL	4 (14,8)	4 (12,9)		4,00 (0,30–53,47)

*Teste Qui-Quadrado;

**Teste Exato de Fisher.

Quando avaliados os distúrbios específicos, a análise mostrou que mulheres (p = 0,021) e pacientes com tempo de tratamento de até 3 anos (p = 0,002) nesse subgrupo apresentaram maior probabilidade de desenvolver TAG. Assim, os homens tiveram 0,28 vezes menor chance de evoluir com TAG (OR = 0,28; IC 95% [0,09–0,84]) e os pacientes com mais de 5 anos em tratamento apresentaram 0,10 vezes menor chance de desenvolver TAG (OR = 0,10; IC 95% [0,02–0,39]).

#### Variáveis Sociodemográficas e Clínicas

Ao se correlacionarem as demais variáveis sociodemográficas e clínicas com a prevalência de Episódio Depressivo Maior, notou-se que nenhuma associação apresentou poder estatístico.

No entanto, ao se avaliar especificamente o TAG, verificou-se que 54% dos pacientes com esse transtorno tinham até 3 anos de tratamento, em comparação a 30,4% dos pacientes sem TAG (p = 0,029). Na análise bivariada, pacientes com > 5 anos de tratamento apresentaram menor risco de desenvolver algum transtorno psiquiátrico (OR = 0,33; IC 95% [0,14–0,77]). Esses dados podem ser visualizados na Tabela 5.

**Tabela 5:** Associação entre dados clínicos e sociodemográficos com Transtorno de Ansiedade Generalizada dos 119 participantes avaliados

Variáveis	Sem TAG n (%)	Com TAG n (%)	p-valor*	OR [I.C. 95%]
Sexo			0,004	
Feminino	22 (31,9)	29 (58,0)		–
Masculino	47 (68,1)	21 (42,0)		0,34 (0,16–0,72)
Idade			0,472	
De 21 a 40 anos	19 (27,5)	9 (18,0)		–
De 41 a 60 anos	32 (46,4)	27 (54,0)		1,78 (0,69–4,58)
≥ 61 anos	18 (26,1)	14 (28,0)		1,64 (0,57–4,72)
Moradia			0,233	
Urbana	55 (79,7)	44 (88,0)		–
Rural	14 (20,3)	6 (12,0)		0,54 (0,19–1,51)
Estado Civil			0,461	
Casado(a)	38 (55,1)	33 (66,0)		–
Solteiro(a)	25 (36,2)	13 (26,0)		0,60 (0,26–1,35)
Viúvo(a)	6 (8,7)	4 (8,0)		0,77 (0,20–2,96)
Tempo de tratamento			0,029	
≤ 3 anos	21 (30,4)	27 (54,0)		–
Entre 3 e 5 anos	17 (24,6)	10 (20,0)		0,46 (0,17–1,20)
≥ 5 anos	31 (44,9)	13 (26,0)		0,33 (0,14–0,77)
KT/V			0,332	
≤ 0,80	12 (17,4)	4 (8,0)		–
Entre 0,81 e 1,20	14 (20,3)	11 (22,0)		2,36 (0,59–9,37)
≥ 1,21	43 (62,3)	35 (70,0)		2,44 (0,72–8,24)
Hemoglobina			0,300	
≤ 9 g/dL	8 (11,6)	2 (4,0)		–
Entre 9,1 e 13 g/dL	50 (72,5)	41 (82,0)		3,28 (0,66–16,30)
≥ 13,1 g/dL	11 (15,9)	7 (14,0)		2,54 (0,41–15,65)

Nota – *Teste Qui-Quadrado.

## Discussão

As dificuldades psicossociais apresentadas pelos pacientes portadores de DRC, particularmente por aqueles em programa crônico de diálise, têm sido alvo de interesse crescente nas últimas décadas. Diversos estudos demonstraram que a alta prevalência de transtornos psiquiátricos nessa população^3,10^ está diretamente relacionada à menor capacidade de manutenção do tratamento dialítico por parte do paciente e a um pior prognóstico, com hospitalizações mais prolongadas e redução da sobrevida dessa população^3,4,11–13^.

Além disso, há uma ênfase cada vez maior na qualidade de vida desses indivíduos, sendo os distúrbios psiquiátricos um dos principais fatores da má qualidade de vida destes pacientes^11–13^. A análise e o estudo destes transtornos nessa população possibilitam a elaboração de estratégias de tratamento específicas, visando ao aumento da sobrevida e à melhoria da qualidade de vida^13^.

Infelizmente, é difícil determinar o real tamanho da prevalência dos transtornos psiquiátricos nesse grupo de pacientes. A possível confusão de sintomas característicos dos transtornos psiquiátricos com sintomas da uremia contribui para esse fato. Irritabilidade, insônia, inapetência, fadiga, perda de libido e dificuldade de concentração são alguns dos sintomas que podem ser atribuídos à ansiedade e à depressão, e que também podem estar presentes no paciente renal crônico por fatores próprios da doença^14^.

Além disso, as toxinas urêmicas contribuem diretamente para danos cerebrais e consequente declínio cognitivo e distúrbios psiquiátricos, assim como alterações hemodinâmicas, anemia, hiperparatireoidismo, polifarmácia e distúrbios do sono^3^.

O uso de questionários em estudos para o diagnóstico dos transtornos é outro fator que pode modificar a real prevalência das doenças, com tendência a superdimensionar seu valor. Esse fato foi observado em um estudo de 1985, no qual a depressão foi observada em 47% dos pacientes em HD após a aplicação do Beck Depression Inventory (BDI). No entanto, após avaliação por métodos mais rigorosos, demonstrou-se que apenas 5% destes pacientes preenchiam os critérios do DSM – III para depressão^11^.

Mesmo considerando a possibilidade de diagnósticos falso-positivos, a detecção de possíveis distúrbios possibilitará maior atenção e empenho por parte da equipe assistente, reparando a subavaliação desses transtornos nos serviços de diálise crônica.

O MINI 5.0.0. é um questionário que possui diversas vantagens para sua utilização. Um dos fatores que corroboraram a decisão por seu uso foi a existência de um estudo prévio, publicado em 2006, avaliando os distúrbios psiquiátricos em pacientes com DRC na Bahia^15^, o que facilita a comparação da prevalência desses distúrbios ao longo dos anos e após a pandemia da COVID-19.

No presente trabalho, uma porcentagem importante da amostra exibiu transtornos psiquiátricos. Isso confirma que altas taxas desses distúrbios ocorrem em unidades de hemodiálise, sendo mais do que o dobro do apontado pelos dados do Ministério da Saúde para a população geral no Brasil, que indicam uma prevalência de transtornos mentais em torno de 20% na população adulta^16^. Em nível internacional, um estudo publicado em 2023, que agregou dados de 29 países e incluiu 156.331entrevistados^17^, demonstrou que a prevalência de transtornos mentais ao longo da vida foi de aproximadamente 29%, sendo os principais o uso abusivo de álcool, o transtorno depressivo maior e a fobia específica^17^.

Já na atual pesquisa, os transtornos mais frequentes encontrados foram os de Episódio Depressivo Maior, atual ou recorrente, em 18,5% dos pacientes, e o Transtorno de Ansiedade Generalizada, em 42%. Esses dados ultrapassam, em muito, as taxas encontradas na população geral latino-americana, estimadas em torno de 8,7% para EDM e 5,5% para TAG^18^. Entre adultos de São Paulo, estudos apontam uma prevalência de 11% para transtornos de humor e 19,9% para transtornos de ansiedade^19^.

No Brasil, dois trabalhos prévios utilizaram o MINI em pacientes renais crônicos. O primeiro deles, realizado há 20 anos, avaliou 244 pacientes dialíticos na Bahia^15^. Esse estudo evidenciou uma prevalência de 37,3% de transtornos psiquiátricos, sendo 8,6% de EDM e 2% de TAG^15^. O outro estudo, de 2009, avaliou apenas os transtornos de humor e psicóticos por meio do MINI. Setenta pacientes em HD do Rio Grande do Sul foram entrevistados e a prevalência de depressão maior encontrada foi de 9,9%^20^.

Existem diversos fatores que podem justificar o aumento da prevalência desses distúrbios ao longo das últimas décadas, como: envelhecimento da população e aumento das doenças crônicas; cobranças externas e auto infligidas elevadas; dificuldade em lidar com situações desafiadoras; exposição crescente à violência e à criminalidade; e o advento da pandemia da COVID-19 e suas consequências^21^.

Mesmo que a utilização de outro método diagnóstico dificulte a comparação com o trabalho atual, vale citar um trabalho realizado nos Estados Unidos em 1993, que avaliou 93% de todos os pacientes dialíticos no país e observou que aproximadamente 9% desses pacientes teve, ao menos, uma internação por diagnóstico primário ou secundário de um transtorno mental no ano estudado^22^. Além disso, uma metanálise avaliou 55.982 pessoas com DRC em todo o mundo, evidenciando uma prevalência entre 22,8% e 39,3%, a depender do método utilizado^10^. Vale ressaltar que esse estudo avaliou sintomas depressivos de maneira geral, e não apenas Episódio Depressivo Maior.

Como citado anteriormente, o advento da pandemia da COVID-19 incrementou ainda mais a alta prevalência dos transtornos psiquiátricos, com taxas de elevação em torno de 27,6% para EDM e de 25,6% para TAG^9,21^. Na vigência da infecção, a prevalência é ainda maior, com 45% dos pacientes apresentando depressão e 47% dos pacientes com ansiedade^9^. Acredita-se que, além dos fatores próprios da infecção e do isolamento social, a preocupação com familiares e amigos, a incerteza quanto ao futuro, as informações incorretas veiculadas nas mídias sociais^23^ e a falta de suprimento médico foram as causas dessa elevação.

Pela suspeita da existência desses diversos contribuintes, e na tentativa de avaliar a contribuição isolada de fatores relacionados à infecção, o presente estudo separou os entrevistados em 2 subgrupos sociodemograficamente semelhantes: aqueles que não tiveram COVID-19 e aqueles que tiveram a infecção. Diferentemente do que se esperava, não foi encontrada associação estatisticamente significativa entre o diagnóstico de COVID-19 e o aumento da prevalência dos distúrbios. Um fator que pode justificar, ao menos em parte, esse achado é o fato de que os pacientes estudados foram aqueles que apresentaram um quadro infeccioso de baixa gravidade. Muitos dos pacientes dialíticos que evoluíram com COVID-19 grave foram a óbito e, portanto, não puderam ser incluídos no presente estudo.

Das variáveis sociodemográficas submetidas à análise, o sexo feminino confirmou dados já descritos na literatura^24–26^, mostrando-se positivamente associado à ocorrência dos transtornos mentais.

A associação foi mais bem evidenciada e estatisticamente significativa quando direcionada ao diagnóstico de TAG ou quando a análise foi feita especialmente no subgrupo de 58 pacientes que tiveram COVID-19. Isso se deve, provavelmente, ao maior número de pacientes com diagnóstico de TAG (50 pacientes), quando comparado com EDM (22 pacientes) e, talvez, ao fato de que, mesmo não havendo associação significativa entre o diagnóstico da COVID-19 e os distúrbios psiquiátricos, a prevalência de mulheres com transtorno psiquiátrico nesse grupo foi maior (58,1% versus 45,2%).

Dessa forma, as mulheres apresentaram o dobro de chance, em relação aos homens, de desenvolverem algum distúrbio psiquiátrico, mais especificamente o TAG. Tais dados reforçam a suspeita de que as mulheres têm maior dificuldade em lidar com situações adversas, além de possuírem traços intrínsecos de personalidade mais suscetíveis a alterações na saúde mental quando confrontadas com problemas crônicos de saúde^26^.

Diferentemente do que sugerem alguns estudos na literatura, não foi encontrada associação entre a moradia, o estado civil e seu consequente suporte familiar^27^. Talvez isso tenha ocorrido porque esse estudo avaliou somente a moradia e o estado civil do paciente, não questionando se ele possuía qualquer tipo de suporte familiar além do cônjuge ou, até mesmo, algum suporte social fora desse âmbito.

No que se refere particularmente à idade, chama atenção o fato de não haver nenhum paciente com menos de 20 anos na amostra, além de a maioria dos pacientes (73,1%) ter entre 20 e 60 anos. Esse perfil epidemiológico é semelhante ao observado em unidades de outros países, onde a maior prevalência, na diálise, é de pacientes entre 20 e 59 anos^28^. No entanto, essa caraterística pode ter contribuído para a falta de associação, uma vez que os idosos apresentam comorbidades que refletem pior qualidade de vida e maior expressão de transtornos psiquiátricos^29,30^. Além disso, alguns estudos mostram que os jovens têm maior dificuldade em lidar com doenças crônicas, apresentando taxas mais elevadas de distúrbios psiquiátricos quando essas doenças, como a DRC, são diagnosticadas^24^.

Outra variável que mostrou associação com os distúrbios psiquiátricos foi o tempo de tratamento da doença. Os pacientes com < 3 anos em hemodiálise apresentaram mais distúrbios, sugerindo que um tempo maior em programa de diálise foi preditor de melhor adaptação ao tratamento, achado consistente com a maioria dos dados de literatura^31^. Um estudo de 1987 demonstrou maior prevalência de depressão nos primeiros 2 anos de tratamento^32^. Pacientes com DRC frequentemente experimentam melhora nas condições médicas após o início da diálise, porém necessitam de algum tempo para se adaptarem psicologicamente à nova situação^31^. Entretanto, não há unanimidade sobre essa questão. Kimmel e cols., em 1998, avaliaram as hospitalizações por doença mental em 176.368 pacientes em diálise. Neste estudo, o tempo em diálise não se mostrou associado a um risco maior de internação^22^.

A presença de anemia não aumentou o risco de os pacientes expressarem transtornos psiquiátricos, apesar de níveis mais elevados de hemoglobina se correlacionarem positivamente com melhor qualidade de vida e melhora dos sintomas^33^. Nossos resultados talvez se devam ao número reduzido de pacientes com hemoglobina inferior a 9g/dL.

Da mesma forma, também não houve associação entre o Kt/V e os transtornos psiquiátricos. Novamente acredita-se que isso se deva ao fato de a maior parte dos pacientes estudados possuírem Kt/V superior a 1,21 (meta almejada)^34^.

### Limitações

Algumas limitações se relacionaram à impossibilidade de avaliação de todas as variáveis de importância que, indubitavelmente, poderiam conferir maior validade ao estudo. Entre elas, as características de personalidade do paciente e sua percepção da doença são reportadas como importantes preditoras de saúde mental^35^, assim como a etiologia da DRC, comorbidades associadas, raça, ocupação, renda familiar, nível educacional, suporte familiar, estado nutricional e alterações hormonais^24,25,31,35^.

Além disso, acredita-se que o tamanho amostral não tenha sido suficiente para conferir significância a algumas diferenças encontradas. Para o cálculo do tamanho amostral, foi utilizada uma prevalência de 50% de distúrbios psiquiátricos, o que foi semelhante à taxa encontrada no presente estudo. No entanto, caso uma prevalência menor fosse utilizada para o cálculo, o tamanho da amostra seria maior, o que traria maior significância aos achados.

A dificuldade na comparação do presente trabalho com outros estudos populacionais também pode ser considerada uma limitação, uma vez que se trata de um estudo de centro único e o questionário MINI utilizado ser uma versão menos atual, ainda baseada nos critérios do DSM-III-R/IV, por ser o único validado para a língua portuguesa^7^.

Deve-se lembrar que existe o risco de confusão de sintomas característicos destes transtornos e fatores orgânicos próprios da DRC^4,14^. No presente estudo, foi realizada a avaliação da efetividade da diálise por meio do Kt/V, e as entrevistas foram feitas após as sessões de hemodiálise, com a intenção de minimizar essa possível confusão. No entanto, sabe-se que existem outras variáveis clínicas e laboratoriais que poderiam contribuir para a avaliação da adequação da diálise e que não foram utilizadas nesse estudo.

Para finalizar, mesmo que a amostra deste estudo seja representativa dos pacientes em HD na Bahia, a comparação com outras unidades do Brasil e do restante do mundo torna-se mais problemática, uma vez que estas apresentam um perfil epidemiológico distinto^36^.

### Implicações Clínicas

Os transtornos psiquiátricos são preditores importantes e independentes de morbidade e mortalidade em diálise, principalmente por estarem associados à não adesão ao tratamento^3,4,11,12^. Em uma metanálise de 2000, com mais de 500 pacientes, os autores concluíram que o risco de não adesão ao tratamento em pacientes deprimidos foi 3 vezes maior do que em pacientes não deprimidos^37^.

Acreditamos que pesquisas sobre aspectos psicossomáticos, psiquiátricos e de qualidade de vida possam exercer uma importante contribuição na tomada de decisões clínicas e de cuidados aos pacientes com DRC. Estudos têm confirmado o papel desses aspectos psicossomáticos em influenciar a morbidade e a mortalidade desses pacientes^13,37^.

Mesmo com a elevada prevalência e a significativa carga clínica e socioeconómica, os transtornos psiquiátricos parecem ser subtratados em pacientes com DRC. Um grande estudo envolvendo 1.099 adultos com DRC que apresentavam sintomas depressivos revelou que apenas 31% dos pacientes relataram ter recebido prescrição de antidepressivos^37^.

Assim como na população geral, o tratamento desses distúrbios deve incluir estratégias não farmacológicas, como psicoterapia, programas de treinamento físico e apoio social^38–40^. Estudos comprovam que programas de exercício físico podem reduzir os sintomas depressivos em pacientes em diálise^38,39^. Um ensaio randomizado com 85 pacientes em hemodiálise e que apresentaram sintomas depressivos demonstrou uma melhora significativa após sessões de 12 semanas de terapia cognitivo-comportamental^40^.

Espera-se que os dados e resultados descritos possam estimular novas pesquisas nesta área, servir de alerta para a altíssima prevalência desses transtornos no âmbito das unidades de diálise e, por fim, contribuir de alguma forma para a melhoria da qualidade de vida e da sobrevida dos pacientes portadores de DRC em programa de hemodiálise.

## Conclusões

A prevalência de transtornos psiquiátricos em pacientes portadores de DRC em programa de hemodiálise crônica, em uma clínica de TRS no interior da Bahia, foi elevada, atingindo 52,1%. Os transtornos mais comuns foram Transtorno de Ansiedade Generalizada e Episódio Depressivo Maior, sendo que a quase totalidade dos pacientes (96,8%) apresentou ao menos um desses dois diagnósticos.

O diagnóstico de COVID-19 não mostrou associação positiva com a ocorrência de qualquer distúrbio psiquiátrico. No entanto, entre o subgrupo de pacientes que tiveram COVID-19, as mulheres apresentaram significativamente mais distúrbios psiquiátricos, enquanto os pacientes com mais de 5 anos de tratamento tiveram uma chance significantemente menor de desenvolver algum transtorno, em comparação aos demais.

## Data Availability

Os conjuntos de dados gerados e analisados durante o presente estudo não estão disponíveis publicamente devido a restrições éticas e de privacidade, mas podem ser obtidos junto ao autor correspondente mediante solicitação razoável.
